# Influenza Vaccination Rates, Awareness, and Attitudes Among Patients With Rheumatoid Arthritis and Systemic Lupus Erythematosus: A Cross-Sectional Study

**DOI:** 10.7759/cureus.85744

**Published:** 2025-06-11

**Authors:** Abdullah H Albin Saad, Mousa J Alhaddad, Mohammed S Almulaify, Hussain A Alwesaibi, Thamer H Alshahrani, Marwan M Alamoudi, Ahmed S Alsalman

**Affiliations:** 1 Internal Medicine, Dammam Medical Complex, Dammam, SAU; 2 Adult Hematology and Hematopoietic Stem Cell Transplantation, King Fahad Specialist Hospital, Dammam, Dammam, SAU; 3 Pulmonary Medicine, Dammam Medical Complex, Dammam, SAU; 4 Rheumatology, Dammam Medical Complex, Dammam, SAU

**Keywords:** influenza vaccine, rheumatoid arthritis, systemic lupus erythematosus, vaccination awareness, vaccination rate

## Abstract

Backgrounds: The influenza vaccine is recommended annually for patients with rheumatoid arthritis (RA) and systemic lupus erythematosus (SLE). This study aimed to report the influenza vaccination rate among RA and SLE patients who are following up at Dammam Medical Complex (DMC), Dammam, Saudi Arabia, to evaluate their understanding of the influenza vaccine and its benefits, and to assess their attitudes regarding it, including any concerns or misconceptions.

Methods: This was a cross-sectional study. It included all patients with RA and SLE who were following up in the rheumatology clinic at DMC. The study was conducted between November 2024 and February 2025. Data was collected through a self-administered paper questionnaire in the clinic. Descriptive statistics, including mean, standard deviation, counts, and percentages, were computed as required. For comparative analyses, the Chi-square test was employed for categorical variables, while the two-sample t-test was used for continuous variables.

Results: A total of 261 patients participated in the study, including 191 RA patients (73.2%) and 70 SLE patients (26.8%). Out of the total, 131 patients (50.2%) reported that they had been vaccinated against influenza in the past. However, only 72 patients (27.6%) confirmed they had received the vaccine within the last two seasons, whereas a significant majority, 189 patients (72.4%), had not been vaccinated during this timeframe. Regarding the perception of the influenza vaccination's safety and effectiveness, 133 patients (51.0%) affirmed that they believed it to be safe and effective. When asked if the seasonal influenza vaccine is the best method to avoid complications from influenza, 153 patients (58.6%) agreed. Most patients (n = 140, 53.6%) were aware that seasonal influenza vaccination is recommended for individuals with chronic diseases.

Conclusions: Our study revealed a low vaccination rate among RA and SLE patients in the last two seasons. To minimize the risk of complications, it is crucial to increase awareness about the benefits of influenza vaccination.

## Introduction

Rheumatoid arthritis (RA) is one of the chronic autoimmune diseases that causes inflammation around the body. It can lead to severe damage to joints and surrounding tissues and can even affect the heart, lungs, and nervous system if left untreated. Although the data are few, the prevalence of RA in Saudi Arabia, at around 0.22%, is believed to be lower than in Western countries [1], with an estimated worldwide prevalence of 0.46% [2].

Systemic lupus erythematosus (SLE) is another chronic autoimmune disease that can have diverse presentations ranging from mild cutaneous manifestations to life-threatening multiorgan involvement, and is associated with morbidity and mortality globally [3], with a local prevalence of 0.19% in Saudi Arabia [4].

Patients with autoimmune inflammatory rheumatic diseases (AIIRDs), such as RA and SLE, are at an increased risk of getting infections secondary to many factors, including the immunosuppressive effect of the disease itself and immunomodulatory medications used for treatment. There is an increased risk of dying from respiratory infections such as pneumonia and influenza in patients with AIIRD. Influenza vaccination showed a reduction of mortality rate and admission rate in elderly patients with AIIRD from influenza or pneumonia [5].

The European League Against Rheumatism (EULAR) recommendations in 2019 established that influenza vaccination should be strongly considered for the majority of patients with AIIRD annually [6], as the vaccine is associated with an 87% risk reduction of influenza infections [7,8].

Our aim in this current study is to determine the rate of influenza vaccination over the past two seasons, and to evaluate the awareness and attitude regarding influenza vaccination among patients with RA and SLE who are following at Dammam Medical Complex (DMC), the largest governmental secondary hospital in the Eastern Province of Saudi Arabia, offering free medical care to the Saudi patients. By focusing on the past two seasons, the study was designed to minimize recall bias that is commonly associated with questionnaire-based studies.

Understanding the vaccination rates in patients with RA and SLE and assessing their awareness and attitudes would contribute to the broader understanding of influenza vaccination in immunocompromised populations, ultimately aiming to reduce morbidity and mortality associated with influenza.

## Materials and methods

This was a cross-sectional study. It included all patients with RA and SLE who were following up in the rheumatology clinic at DMC. The study was conducted between November 2024 and February 2025. Data was collected through a self-administered paper questionnaire in the clinic. The questionnaire was adopted from a questionnaire used in two previous studies [9,10], with adding questions related to the use of the influenza vaccine in pregnancy, its cost in Saudi Arabia, and whether the participants received prior physician counseling about it. Translation of the questionnaire from English to Arabic was done by the authors. The authors then compared the translated questionnaire to the original questionnaire to ensure that the intents of the questions were preserved (see Appendix A).

The inclusion criteria were composed of all patients who were 18 years old and older, who were diagnosed to have RA and SLE, and who visited the clinics during the study period. For the exclusion criteria, the patients who could not read Arabic were excluded from the study. The study was reviewed by the Institutional Review Board (IRB) at DMC on November 4, 2024 (approval number: IM-38).

Data analysis was performed using Python (version 3.12.7; Python Software Foundation, USA) in conjunction with the SciPy library (version 1.13.1). Descriptive statistics, including mean, standard deviation, count, and percentage, were computed as required. For comparative analyses, the Chi-square test was employed for categorical variables, while the two-sample t-test was used for continuous variables. A p-value of <0.05 was considered statistically significant.

## Results

A total of 288 questionnaires were distributed, and 261 (90.6%) were completed by the participants, including 191 RA patients (73.2%) and 70 SLE patients (26.8%). The mean age of the participants was 48.04 ± 13.87 years. Most patients were female (n = 230, 88.12%), while 31 patients (11.88%) were male. The majority of patients were Saudi (n = 247, 94.6%), with a small proportion being non-Saudi (n = 14, 5.4%). In terms of education level, 109 patients (41.8%) had completed university education, 81 patients (31.0%) had a high school education, and 71 patients (27.2%) had an education below high school. The patients' demographics are shown in Table 1.

**Table 1:** Patients' Demographics (n = 261) SLE: systemic lupus erythematosus; RA: rheumatoid arthritis

Characteristics		n (%)
Age (Mean ± SD, years)		48.04±13.87
Gender	Male	31 (11.88)
	Female	230 (88.12)
Nationality	Saudi	247 (94.64)
	Non-Saudi	14 (5.36)
Education level	University	109 (41.76)
	High school	81 (31.03)
	Below high school	71 (27.2)
Diagnosis	SLE	70 (26.82)
	RA	191 (73.18)

Out of the total, 131 patients (50.2%) reported that they had been vaccinated against influenza in the past, while 130 patients (49.8%) indicated they had not received the vaccine. However, only 72 patients (27.6%, 95% confidence interval (CI): 21.5% to 31.7%) confirmed they had received the vaccine within the last two seasons, whereas a significant majority, 189 patients (72.4%), had not been vaccinated during this timeframe. Additionally, regarding prior information about the vaccine from a physician, 149 patients (57.1%) stated they had been informed, while 112 patients (42.9%) reported they had not received any information from a healthcare provider. The influenza vaccination rates of the patients are presented in Table 2.

**Table 2:** Vaccination Characteristics of Study Participants (n = 261)

Characteristics		n (%)
Age (Mean ± SD, years)		48.04±13.87
Vaccinated before	Yes	131 (50.19)
	No	130 (49.81)
Vaccinated in the last two seasons	Yes	72 (27.59)
	No	189 (72.41)
Informed before about the vaccine by a physician	Yes	149 (57.09)
	No	112 (42.91)

Regarding the perception of the influenza vaccination's safety and effectiveness, 133 patients (51.0%) affirmed that they believed it to be safe and effective, while 84 patients (32.2%) were unsure, and 44 patients (16.9%) disagreed. When asked if the seasonal influenza vaccine is the best method to avoid complications from influenza, 153 patients (58.6%) agreed. Conversely, 57 patients (21.8%) expressed uncertainty, and 51 patients (19.5%) disagreed with this statement. Most patients (n = 140, 53.6%) were aware that seasonal influenza vaccination is recommended for individuals with chronic diseases. However, only 24.9% (n = 65) of patients knew that pregnant women can be vaccinated against influenza. Concerning the belief that the seasonal influenza vaccine weakens the immune system, 118 patients (45.2%) disagreed, while 87 patients (33.3%) were unsure, and 56 patients (21.5%) believed it could weaken immunity. A significant proportion of patients (45.6%, n = 119) believed that herbal and traditional medicines are better than seasonal influenza vaccination. Almost all patients (99.2%, n = 259) were aware that the seasonal influenza vaccine is freely provided in Saudi Arabia. The patients' knowledge, awareness, and attitudes regarding influenza vaccines are described in Table 3.

**Table 3:** Patients' Knowledge, Awareness, and Attitudes Regarding Influenza Vaccines (n = 261)

Characteristics		n (%)
The influenza vaccination is safe and effective	Yes	133 (50.96)
	Not sure	84 (32.18)
	No	44 (16.86)
The best way to avoid the complications of influenza is by using the seasonal vaccine	Yes	153 (58.62)
	Not sure	57 (21.84)
	No	51 (19.54)
Seasonal influenza is recommended to be given to individuals with chronic diseases	Yes	140 (53.64)
	Not sure	81 (31.03)
	No	40 (15.33)
Pregnant women can be vaccinated with the influenza vaccine	Yes	65 (24.9)
	Not sure	147 (56.32)
	No	49 (18.77)
Seasonal influenza vaccine weakens the immune system and renders it susceptible to infections	No	118 (45.21)
	Not sure	87 (33.33)
	Yes	56 (21.46)
The herbal and traditional medicines are better than seasonal influenza vaccination	No	98 (37.55)
	Not sure	44 (16.86)
	Yes	119 (45.59)
The seasonal influenza vaccine is freely provided in Saudi Arabia	Yes	259 (99.23)
	No	2 (0.77)

The mean age of patients with SLE was significantly lower than that of patients with RA (40.46 ± 13.76 vs. 50.82 ± 12.87 years, p < 0.001). There were no significant differences between the two groups in terms of sex, perception of the safety and effectiveness of the influenza vaccine, and attitudes regarding it. However, patients with SLE were more likely to believe that pregnant women can be vaccinated with the influenza vaccine (38.6% vs. 19.9%, p = 0.003). The detailed comparison of the influenza vaccination-related knowledge, attitudes, and rates between patients with SLE and RA is presented in Table 4.

**Table 4:** Comparison Between Systemic Lupus Erythematosus (SLE) and Rheumatoid Arthritis (RA) Patients (n =261) * The Chi-square test was used to compare categorical variables, while the two-sample t-test was used for continuous variables. A p-value of <0.05 was considered to indicate statistical significance.

Characteristics	SLE (n = 70)	RA (n = 191)	P value
Age, mean ± SD, years	40.46±13.76	50.82±12.87	0*
Male, n (%)	6 (8.57)	25 (13.09)	0.433
The influenza vaccination is safe and effective, n (%)	29 (41.43)	104 (54.45)	0.085
The best way to avoid the complications of influenza is by using the seasonal vaccine, n (%)	37 (52.86)	116 (60.73)	0.316
Seasonal influenza is recommended to be given to individuals with chronic diseases, n (%)	39 (55.71)	101 (52.88)	0.790
Pregnant women can be vaccinated with the influenza vaccine, n (%)	27 (38.57)	38 (19.9)	0.003*
Seasonal influenza vaccine weakens the immune system and renders it susceptible to infections, n (%)	15 (21.43)	41 (21.47)	1
The herbal and traditional medicines are better than seasonal influenza vaccination, n (%)	31 (44.29)	88 (46.07)	0.907
The seasonal influenza vaccine is freely provided in Saudi Arabia, n (%)	69 (98.57)	190 (99.48)	1
Vaccinated before, n (%)	29 (41.43)	102 (53.4)	0.115
Vaccinated in the last two seasons, n (%)	13 (18.57)	59 (30.89)	0.069
Informed before about the vaccine by a physician, n (%)	42 (60.0)	107 (56.02)	0.664

The patients who received a prior influenza vaccine were significantly older than those who were never vaccinated (49.75 ± 14.31 vs. 46.32 ± 13.25 years, p < 0.05). There were no significant differences between the two groups in terms of sex, nationality, or education level. The patients who received a prior influenza vaccine were more likely to believe that the influenza vaccination is safe and effective (66.4% vs. 35.4%, p < 0.001), that the best way to avoid influenza complications is by using the seasonal vaccine (68.7% vs. 48.5%, p < 0.01), and that seasonal influenza is recommended for individuals with chronic diseases (65.7% vs. 41.5%, p < 0.001). Additionally, the patients who received a prior influenza vaccine were more likely to have been informed about the vaccine by a physician (64.9% vs. 49.2%, p < 0.05). The detailed comparison between the patients who received a prior influenza vaccine and those who were never vaccinated is shown in Table 5.

**Table 5:** Comparison Between Patients Who Received a Prior Influenza Vaccine and Those Who Were Never Vaccinated (N = 261) * The Chi-square test was used to compare categorical variables, while the two-sample t-test was used for continuous variables. A p-value of <0.05 was considered to indicate statistical significance.

Characteristics	Vaccinated (n = 131)	Never Vaccinated (n = 130)	P value
Age, mean ± SD, years	49.75±14.31	46.32±13.25	0.046*
Male, n (%)	18 (13.74)	13 (10.0)	0.458
Non-Saudi, n (%)	8 (6.11)	6 (4.62)	0.795
University Education, n (%)	53 (40.46)	56 (43.08)	0.762
The influenza vaccination is safe and effective, n (%)	87 (66.41)	46 (35.38)	0*
The best way to avoid the complications of influenza is by using the seasonal vaccine, n (%)	90 (68.7)	63 (48.46)	0.001*
Seasonal influenza is recommended to be given to individuals with chronic diseases, n (%)	86 (65.65)	54 (41.54)	0*
Pregnant women can be vaccinated with the influenza vaccine, n (%)	39 (29.77)	26 (20.0)	0.093
Seasonal influenza vaccine weakens the immune system, and renders it susceptible to infections, n (%)	31 (23.66)	25 (19.23)	0.471
The herbal and traditional medicines are better than seasonal influenza vaccination, n (%)	53 (40.46)	66 (50.77)	0.121
The seasonal influenza vaccine is freely provided in Saudi Arabia, n (%)	130 (99.24)	129 (99.23)	1
Informed before about the vaccine by a physician, n (%)	85 (64.89)	64 (49.23)	0.015*

## Discussion

We found in the present study that only 27.6% of patients with RA and SLE had received the influenza vaccine in the last two seasons, while around half of the patients had never been vaccinated, putting them at high risk of complications. This low percentage of vaccination indicates underutilization of one of the safest and most effective ways to prevent influenza complications in such patients and it contrasts with EULAR’s 2019 recommendations regarding influenza vaccination which strongly recommends taking the vaccine for patients with AIIRD even when treated with disease-modifying antirheumatic drugs such as infliximab, etanercept, or adalimumab [6].

A Mexican study in patients with rheumatic diseases showed that 79.8% of the patients were vaccinated for influenza at least once [10]. In Saudi Arabia and the other Gulf Cooperation Council (GCC) countries, such information is lacking. However, the influenza vaccination rates were reported in other patients with non-rheumatic diseases. As an example, a study done at the security forces hospital in Riyadh for patients with diabetes mellitus (DM) regarding the prevalence of influenza vaccination showed that only 47.8% had taken the vaccine [11]. Another study that was also conducted in Riyadh showed that only 15.6% had taken the vaccine in the same year of the study, and only 8.8% of the participants were taking the vaccine every year [12]. Another study in Qatar for patients with DM showed that only 21.3% of the patients have received the vaccine [13]. Multiple barriers were contributing to these low vaccination rates, including fearing vaccine side effects [14,15], believing that the vaccine is unnecessary [16,17], and being unaware of vaccine availability [18].

Overall, our study found a higher vaccination rate compared to some studies conducted outside the GCC countries. In southwest China, a study has been done that showed that influenza vaccination among patients with SLE was only 8.3% [19]. Another study was done in Islamabad, and it showed that the influenza vaccination rate in patients with RA was only 3% [20]. Also, there is a study done in Tunisia for elderly patients with chronic diseases, which showed that the influenza vaccination rate in the 2018-2019 season was only 19.4% [21].

Regarding awareness and attitude related to influenza vaccine among patients with RA and SLE, approximately half of the participants, 49.04%, did not believe or were not sure if the influenza vaccine is safe and effective, which may be one of the explanations for the low vaccination rate in the last two seasons of influenza.

We found that the patients who were vaccinated before believe that the vaccine is safe and effective, know that it is recommended to be given to individuals with chronic diseases, and are aware that it is the best way to avoid complications of influenza, more than the patients who were not vaccinated before. This indicates that the awareness level is important and that the lack of awareness leads to underutilization of the vaccine. Also, physician input regarding influenza vaccination plays a major role in the awareness and attitude among the participants.

To increase the rate of influenza vaccination among patients with rheumatological diseases, it is important to increase the awareness level of the patients regarding the vaccine and its benefits in reducing the risk of complications, including the risks of hospitalization, morbidity, and mortality. Also, collaboration between primary health care, secondary hospitals, and tertiary hospitals regarding vaccination can increase the rate of vaccination.

The limitations of our study include that the data have been collected in a single center, which limits the generalizability of the results to other centers and other regions in Saudi Arabia. The fact that the majority of the patients being treated at DMC are Saudi citizens reflects that the study might be especially unrepresentative of the non-Saudi residents in Saudi Arabia. In addition, the study included only the patients who could read Arabic. Therefore, it would not be representative of the non-Saudi residents. Finally, the data was collected through a self-administered questionnaire in a cross-sectional study design, which can introduce recall biases and does not allow for proving temporal and causal relationships.

## Conclusions

Our study found a low influenza vaccination rate among patients with RA and SLE in the past two seasons. Notably, patients who had previously received the influenza vaccine demonstrated higher awareness levels, suggesting that awareness significantly influences vaccine acceptance. To mitigate the risk of influenza complications, enhancing awareness about the benefits and necessity of vaccination among autoimmune disease patients, particularly those on immunosuppressive therapy, is essential. To enhance this awareness, multiple strategies could be proposed to be studied and tried, including public health campaigns, social media utilization, text message reminders, and healthcare provider education. The latter is notably important for the observation that vaccinated patients were more likely to have received physician counseling, highlighting the importance of healthcare providers taking a proactive educational role in promoting vaccination during patient encounters.

## Appendices

Appendix A

Arabic Translation of the Questionnaire

The questionnaire was translated into Arabic from the original version [9,10], which is licensed under Creative Commons Attribution 4.0 International (CC BY 4.0). No additional permission was required for translation or use.

أنت مدعو للمشاركة في هذا البحث الذي يهدف الى دراسة (المعدل والوعي والإدراك للقاح الإنفلونزا تجاه مرضى التهاب المفاصل الروماتويدي والذئبة في مجمع الدمام الطبي).

إجابتك تعني الموافقة للانضمام لهذا البحث والذي يتضمن الإجابة على أسئلة هذا الاستبيان.

إجابتك سوف تكون سرية ولن يطلع عليها أحد ولن يكتب اسمك بالاستبيان.

مشاركتك في الاستبيان اختيارية ولك مطلق الحرية في التوقف عن الإجابة على الاستبيان في أي وقت.

رفضك للمشاركة أو توقفك عن الإجابة لن يترتب عليه أي عواقب.

تعبئة الاستبيان تستغرق (5 دقائق)، نقدر وقتك وتعاونك.

العمر

........................................ سنة.

الجنس

1) ذكر.

2) أنثى.

الجنسية

1) سعودي

2) غير سعودي

المستوى التعليمي

1) أقل من ثانوي.

2) ثانوي.

3) جامعي.

التشخيص

1) مرض التهاب المفاصل الروماتويدي.

2) مرض الذئبة.

س1) هل تعتقد أن تطعيم الانفلونزا الموسمية آمن وفعال ؟

1) نعم.                           2) لا.                      3) لا أعلم.

س2) هل تعتقد أن أفضل طريقة لتقليل مضاعفات الإنفلونزا الموسمية هو عن طريق أخذ لقاح الإنفلونزا؟

 1) نعم.                           2) لا.                      3) لا أعلم.

س3) هل بإعتقادك أن لقاح الإنفلونزا الموسمية موصى لمن  لديهم أمراض مزمنة (تشمل الامراض الروماتيزمية والمناعية) ؟

1) نعم.                           2) لا.                      3) لا أعلم.

س4) هل تستطيع المرأه الحامل الحصول على لقاح الانفلونزا الموسمية ؟

1) نعم.                           2) لا.                      3) لا أعلم.

س5) هل بإعتقادك ان لقاح الانفلونزا الموسمية يضعف الجهاز المناعي ويجعل الشخص عرضة أكثر للعدوى ؟

1) نعم.                           2) لا.                      3) لا اعلم.

س6) هل تعتقد ان العلاجات الشعبية او التقليدية او بعض الأغذية مثل (البرتقال) أفضل من لقاح الانفلونزا الموسمية ؟

1) نعم.                           2) لا.                      3) لا أعلم.

س7) هل تعلم بأن لقاح الإنفلونزا يعطى مجانا في المملكة العربية السعودية ؟

1) نعم.                           2) لا.              

س8) هل حصلت على تطعيم الإنفلونزا الموسمية من قبل؟

1) نعم.                           2) لا.                

س9) هل حصلت على تطعيم الإنفلونزا في الموسمين السابقين للإنفلونزا الموسمية  2022 و 2023 ؟

1) نعم.                           2) لا.     

س10) هل تم اخبارك عن تطعيم الإنفلونزا الموسمية من قبل طبيبك سابقا؟

1) نعم.                           2) لا.     
